# The association between carotid intima-media thickness and new-onset hypertension in a Chinese community-based population

**DOI:** 10.1186/s12872-019-1266-1

**Published:** 2019-11-27

**Authors:** Long Zhang, Fangfang Fan, Litong Qi, Jia Jia, Ying Yang, Jianping Li, Yan Zhang

**Affiliations:** Department of Cardiology, Peking University First Hospital, No. 8 Xishiku St, Xicheng District, Beijing, 100034 China

**Keywords:** Carotid intima-media thickness, Incidence of hypertension, Chinese population

## Abstract

**Background:**

Hypertension and arterial vasculopathy may be mutual causes and effects. It is unknown whether carotid intima-media thickness (cIMT) is reliably predictive of the presence of newly developed hypertension in the Chinese population. This study evaluated the impacts of cIMT on new-onset hypertension in a community-based population without hypertension at baseline in China.

**Methods:**

A total of 672 Chinese subjects who had complete data for demographics, baseline and follow-up blood pressure measurements, and cIMT measurements at baseline were included in our study. Baseline cIMT was obtained under standardized procedures using the GE Vivid 7 ultrasound system equipped with an 8-MHz linear array vascular probe (GE Medical Systems, Milwaukee, Wl, USA). The outcome was the incidence of hypertension at follow-up. Multivariate regression models were used to access the association between baseline cIMT and the risk of new-onset hypertension.

**Results:**

Subjects were 51.5 ± 4.7 years old, and 32.0% were male. The mean baseline systolic blood pressure (SBP) was 122.5 ± 10.0 mmHg. The mean baseline diastolic blood pressure (DBP) was 72.4 ± 7.5 mmHg. The number of subjects with thickened cIMT (maximum ≥0.9 mm) at baseline was 198 (29.5%). After 2.3 years of follow-up, the rate of new-onset hypertension was 12.6%. The incidence rates of hypertension in the groups with thickened cIMT and normal cIMT were 19.2 and 9.9%, respectively. In the multivariable logistic regression analyses, both the average (OR = 1.69, 95% CI: 1.30–2.19, *P* = 0.0001) and maximum (OR = 1.55, 95% CI: 1.23–1.95, *P* = 0.0002) cIMT were significantly associated with new-onset hypertension after adjustment for various confounders. The group with thickened cIMT showed a higher risk for the incidence of hypertension, with an OR of 1.82 (95% CI: 1.07–3.10, *P* = 0.0270), compared to the normal group.

**Conclusion:**

Thickened cIMT has a strong association with incident hypertension risk in a community-based population without hypertension at baseline in China.

## Background

Hypertension is a serious challenge of global public health and is recognized as an important factor for increased risk of cerebrovascular disease, cardiovascular disease(CVD), peripheral vascular disease, and mortality [1–4]. High systolic blood pressure(SBP) was associated with the highest burden among risk factors from the Global Burden of Disease, Injuries, and Risk Factor study in 2015 [5]. However, the pathogenesis of essential hypertension remains unclear. Changes in arterial structure and function are possible causes of elevated blood pressure.

Carotid intima-media thickness (cIMT) is a simple and convenient, noninvasive indicator reflecting changes in vascular structure and is currently widely used in CVD risk assessment [6]. Previous studies have found that cIMT was significantly associated with cardiovascular outcomes [7–9]. Meanwhile, traditional cardiovascular risk factors, including elderly age, hypertension, diabetes, dyslipidaemia, smoking, etc., have been shown to lead to an increase in cIMT [10–12]. Among them, the relationship between blood pressure and cIMT is particularly close: a large number of cross-sectional studies have suggested that elevated blood pressure shows close correlation with the increase in cIMT in people with and without hypertension [13–15]. At present, thickened cIMT has been considered one of the factors of atherosclerotic preclinical lesions and the target organ that incurs damage due to hypertension. Additionally, thickened cIMT could still be detected in nonhypertensive people, suggesting that there may be some other risk factors that contribute to cIMT thickening in this portion of the nonhypertensive population. These factors may also cause hypertension and CVD. On the other hand, a significant increase in the thickness of the arterial wall may promote a further increase in blood pressure by some possible mechanisms, such as leading to vascular endothelial dysfunction and the stretching effect of the baroreceptors in the aorta and carotid body. Therefore, we hypothesize that cIMT can be used as a factor that reflects the risk of new-onset hypertension in a nonhypertensive population. A previous study confirmed that indicators reflecting arterial stiffness were associated with the incidence of hypertension [16]. It is unknown whether cIMT, which reflects changes in vascular structure, shows a strong association with the presence of newly developed hypertension. This study, using a community-based cohort in China, aims to understand the relationship between cIMT and new-onset hypertension and provide possible help in exploring the underlying mechanisms of hypertension.

## Methods

### Patient enrolment

The community-based cohort was set up in the Beijing Gucheng and Pingguoyuan communities in China between December 2011 and April 2012 as described previously [17]. In brief, baseline survey was conducted in a total of 9540 residents aged ≥40 years, and thena follow-up survey was organized in 5962 subjects with exome chip genotypes from May 2014 to July 2014. A total of 3823 (64.1%) of them took part in the follow-up on site but 1896 participants were excluded since they had a diagnosis of hypertension at baseline. We further excluded participants without cIMT measurement at baseline (1255). Finally, a total of 672 eligible subjects with a mean 2.3 years follow-up were included in this analysis. This study was approved by the ethics committee of Peking University First Hospital. All subjects had signed the informed consent.

### Data collection

Data were collected according to standard operating procedures described in the previous study [17]. We collected height (kg) and weight (m) to calculate the Body mass index (BMI). The information such as sociodemographic status and medical history were obtained from standardized questionnaires. The habits of smoking and drinking were also collected.

The peripheral (brachial) blood pressure of every participant was measured by the Omron (Kyoto, Japan) HEM-7117 electronic blood pressure monitor after a 5-min rest. Each patient’s blood pressure was totally measured three times with ≥1 min interval. Both SBP and diastolic blood pressure (DBP) values were calculated as the mean of three consecutive measurements in our analysis.

Baseline cIMT was obtained according to the standardized protocol using a GE Vivid 7 ultrasound system (GE Medical Systems, Milwaukee, Wl, USA) equipped with an 8-MHz linear array vascular probe by staffs with extensive operational experience. Following the Mannheim Carotid Intima-Media Thickness and Plaque Consensus [18], cIMT was measured in segments that avoid plaque sites. We chose to measure the thickness of the carotid intima-media in the common carotid artery (CCA) on both the left and right sides. The measurement range included a length of 10 mm near the bulb on both sides of the CCAs. The software can automatically track the measurement of the distal wall cIMT, recording the maximum, minimum and average cIMT along the CCAs. cIMT thickening was defined as a maximum thickness ≥ 0.9 mm. The mean cIMT was calculated by the average cIMT value in the systolic phase of the left and right sides of the CCAs.

### Definitions of hypertension

Hypertension was defined as a SBP ≥140 mmHg and/or a DBP ≥90 mmHg and/or a history of hypertension and/or the use of antihypertensive medication.

### Statistical analysis

Mean ± Standard deviation(SD) were used to describe the continuous variables, and percentages (%) were used to describe the categorical variables. The t-test for comparison the continuous variables with normally distributed. For categorical variables, we used the χ2 test. A generalized additive model using the spline smoothing function was used to explore the relationship between cIMT and incident hypertension with adjustments for potential confounders. Covariables were selected based on previous studies that presented a relationship to hypertension [1, 2, 5], including gender, age, BMI, SBP, current smoking and drinking status, dyslipidaemia, diabetes mellitus, CVD history, antidiabetic medications, hypolipidaemic medications and baseline estimated glomerular filtration rate(eGFR). Logistic regression models were used to explore the predictive effect between cIMT and the occurrence of new-onset hypertension in multivariate analyses. We put cIMT into the model using the average and maximum values and whether the cIMT was thickened. We had three sets of multivariable models for examining the relationship between cIMT and incident hypertension risk: the first one did not adjust for other variables, the second one adjusted for age and gender, and the third one further adjusted for the same variables as the smooth curve. All analyses were performed using Empower(R) (www.empowerstats.com, X&Y solutions Boston, MA, USA) and R (http://www.R-project.org). A *p* value < 0.05 was considered statistically significant.

## Results

### Participant characteristics

A total of 672 Chinese individuals without hypertension at baseline were incorporated into the study. The baseline characteristics of all participants are shown in Table 1. Participants were 51.5 ± 4.7 years old, and 32.0% were male. The baseline (mean [SD]) SBP was 122.5 ± 10.0 mmHg, the DBP was 72.4 ± 7.5 mmHg, and the BMI was 25.2 ± 3.2 kg/m^2^. A total of 21.0% of the subjects were currently smoking, while 27.1% were currently drinking. Diabetes was present in 13.2% (*n* = 89) of all participants. A total of 67.1% (*n* = 451) of the participants had dyslipidaemia, and another 3.6% (*n* = 24) had CVD.

**Table 1:** Baseline characteristics of the study population, overall and according to cIMT status at baseline. pSBP, peripheral systolic blood pressure; pDBP, peripheral diastolic blood pressure; eGFR, estimated glomerular filtration rate

Variable	Total study population	cIMT < 0.9 mm	cIMT ≥ 0.9 mm	*P* Value
	*n* = 672	474	198	
Age, mean ± SD, y	51.5 ± 4.7	50.9 ± 4.7	52.9 ± 4.4	< 0.001
Men, n (%)	215 (32.0)	127 (26.8)	88 (44.4)	< 0.001
Body mass index, mean ± SD, kg/m^2^	25.2 ± 3.2	24.8 ± 3.2	26.0 ± 3.1	< 0.001
pSBP, mean ± SD, mmHg	122.5 ± 10.0	121.7 ± 10.1	124.5 ± 9.4	0.001
pDBP, mean ± SD, mmHg	72.4 ± 7.5	72.2 ± 7.4	72.9 ± 7.7	0.179
eGFR, mean ± SD, mL/min per 1.73 m^2^	99.6 ± 9.8	100.7 ± 9.3	96.9 ± 10.5	< 0.001
Current smoking, n (%)	141 (21.0)	82 (17.3)	59 (29.8)	< 0.001
Current drinking, n (%)	182 (27.1)	117 (24.7)	65 (32.8)	0.030
Dyslipidaemia, n (%)	451 (67.1)	308 (65.0)	143 (72.2)	0.068
Diabetes mellitus, n (%)	89 (13.2)	50 (10.5)	39 (19.7)	0.001
Cardiovascular disease, n (%)	24 (3.6)	10 (2.1)	14 (7.1)	0.003
Lipid-lowering medication, n (%)	34 (5.1)	21 (4.5)	13 (6.6)	0.251
Hypoglycaemic medication, n (%)	35 (5.2)	19 (4.0)	16 (8.1)	0.029

The number of subjects with thickened cIMT was 198 (29.5%) at baseline. The participants with thickened cIMT at baseline were older and presented higher BMI; SBP; rates of currently smoking and drinking; prevalence of diabetes, dyslipidaemia and CVD; and rate of hypoglycaemic agent use.

### Association between cIMT and new-onset hypertension

**Fig. 1:**
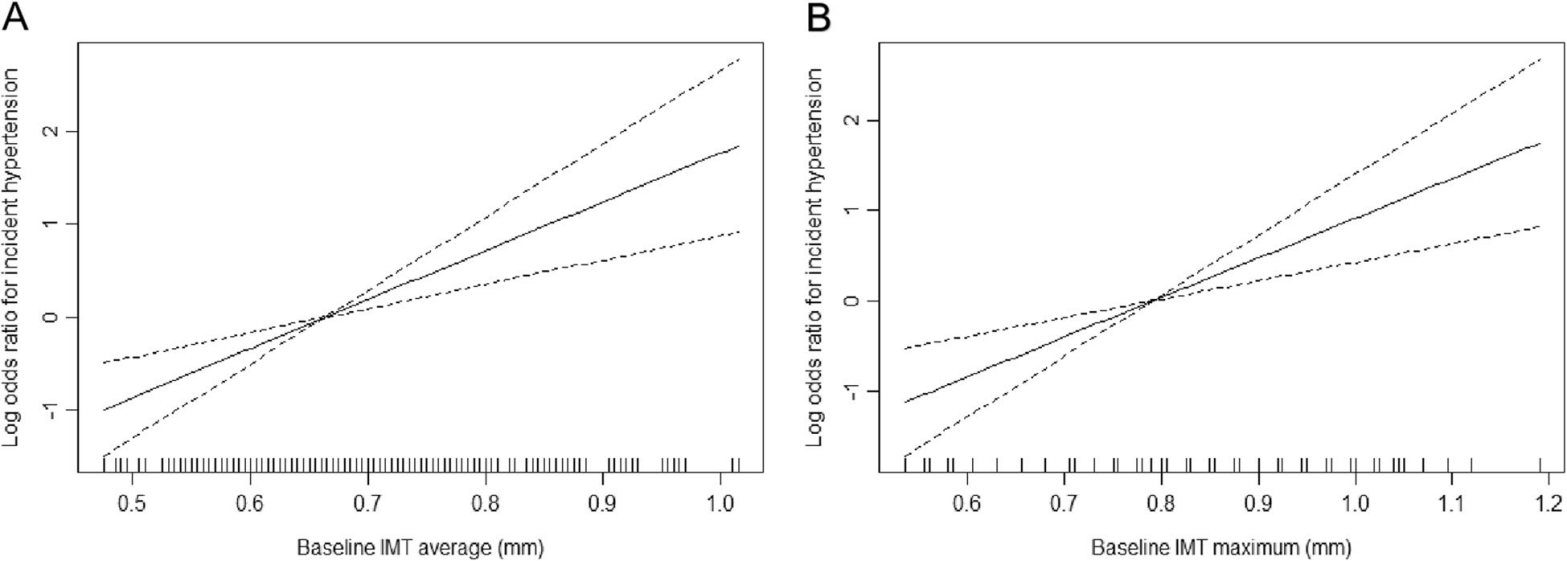
Multivariable adjusted spline curves for relation between the average (**a**) and maximum (**b**) cIMT values and the new-onset hypertension. The dotted line indicates the 95% confidence interval. The inside marks along with x-axis indicates data density. Adjust for sex, age, BMI, SBP, current smoking and drinking status, dyslipidaemia, diabetes mellitus, history of cardiovascular disease, lipid-lowering medications, hypoglycaemic medications and baseline eGFR

After a mean 2.3-year follow-up (median: 2.34, 25th percentile–75th percentile: 2.28–2.39), 12.6% (*n* = 85) of subjects suffered from hypertension. The results of the smooth curve reflecting the relationship between cIMT and incident hypertension are shown in Fig. 1. Both the average and maximum cIMT show a positive linear correlation with incident hypertension.

The results of the multivariate regression for the association of cIMT with new-onset hypertension are shown in Table 2. In the model with no adjustment, both the average (odds ratio [OR] = 1.83, 95% confidence interval [CI]: 1.46–2.29, *P* < 0.0001) and maximum (OR = 1.68, 95% CI: 1.38–2.05, *P* < 0.0001) values of cIMT were significantly associated with new-onset hypertension. The relationship remained statistically significant in model 3 after adjusting for various baseline parameters, with ORs of 1.69 (95% CI: 1.30–2.19, *P* = 0.0001) and 1.55 (95% CI: 1.23–1.95, *P* = 0.0002), respectively.

**Table 2:** Multivariate analysis of the association between cIMT and new-onset hypertension. ^a^Model 2: adjust for age and sex. ^b^Model 3: adjust for sex, age, BMI, SBP, current smoking and drinking status, dyslipidaemia, diabetes mellitus, history of cardiovascular disease, lipid-lowering medications, hypoglycaemic medications and baseline eGFR

Variable	n	Incidence of hypertension	Non-Adjusted Model 1		Multivariate-Adjusted Model 2^a^		Multivariate-Adjusted Model 3^b^	
		n(%)	OR (95% CI)	*P*	OR (95% CI)	*P*	OR (95% CI)	*P*
Average cIMT (per 0.1 mm)			1.83 (1.46, 2.29)	< 0.0001	1.84 (1.45, 2.34)	< 0.0001	1.69 (1.30, 2.19)	0.0001
Max of cIMT (per 0.1 mm)			1.68 (1.38, 2.05)	< 0.0001	1.70 (1.37, 2.09)	< 0.0001	1.55 (1.23, 1.95)	0.0002
cIMT< 0.9 mm	474	47 (9.9)	1		1		1	
cIMT≥0.9 mm	198	38 (19.2)	2.16 (1.36, 3.43)	0.0012	2.04 (1.26, 3.31)	0.0037	1.82 (1.07, 3.10)	0.0270

Furthermore, we divided all participants into two groups according to whether the baseline cIMT was thickened. A total of 19.2% of the subjects in the thickened cIMT group suffered from hypertension at follow-up. We noticed that the group with thickened cIMT showed a significantly higher risk for the incidence of hypertension, with an OR of 2.16 (95% CI: 1.36–3.43, *P* = 0.0012) compared with the group with normal cIMT in the model without adjusting variables. Consistently, the full model that adjusted the baseline parameters still showed a significant association between thickened cIMT and the incidence of hypertension (OR = 1.82, 95% CI: 1.07–3.10, *P* = 0.0270).

## Discussion

In our study, based on a Chinese community population without hypertension at baseline, we identified that both the higher average and maximum cIMT were independently associated with a higher incidence of hypertension. The group with thickened cIMT (maximum ≥0.9 mm) showed a significantly higher risk for new-onset hypertension. The study suggested that changes in arterial structure may be a possible underlying mechanism leading to hypertension.

A number of previous studies have confirmed that there is a close relationship among cIMT, hypertension and other blood pressure indicators [13–15, 19–21], and these studies are mostly cross-sectional. In the study of Ferreira JP et al. [13], individuals with higher cIMT (> 0.9 mm) showed a significant association with the history of hypertension, with an OR of 2.11 after adjusting for other variables. This study also presented a significant correlation between SBP and the risk of cIMT> 0.9 mm, and this correlation was not affected by the history of hypertension (P for interaction > 0.10). A previous study by our team demonstrated that the correlation between blood pressure and cIMT was more significant in a cIMT-thickened population [22]. This result suggested that thickening of cIMT may occur before overt hypertension.

Carotid intima-media thickness is one of the target organs damaged by hypertension, reflecting the injury of vascular structure, while pulse wave velocity (PWV) is considered a factor that reflects vascular dysfunction. Some previous studies have focused on investigating the role of PWV in predicting new-onset hypertension. The Baltimore Longitudinal Study of Aging [16] demonstrated that PWV was an independent predictor of incident hypertension, with a hazard ratio (HR) of 1.10 per 1 m/s increase in PWV after a median follow-up of 4.3 years. Kaess BM et al. [23] also elaborated similar results in their study that an increase in arterial stiffness preceded by hypertension may be an important cause of elevated blood pressure. However, few studies have focused on the role of cIMT in reflecting the changes in vascular structure with new-onset hypertension.

Our study found that the risk of incident hypertension in the thickened cIMT group was significantly higher than that in the normal cIMT group after a mean 2.3-year follow-up. This is the first study to clarify the relationship between cIMT and new-onset hypertension in a Chinese population. The longitudinal study based on the Multiethnic Study of Atherosclerosis [24] demonstrated that increased maximum common cIMT was independently associated with the incidence of hypertension starting at the fourth quintile after adjustment for other variables compared with the lowest quartile after a mean 4.3-year follow-up. Recently, a study from Korea [25] that focused on the predictive value of target organ damage for incident hypertension showed that higher cIMT (per 1-SD increment) was associated with an HR of 1.42 (*P* < 0.001) for incident hypertension after a follow-up period of 4 years. These findings are consistent with the results of our study. Compared with previous studies, our study was shorter in follow-up time and explored the predicting effect of average and maximum cIMT and whether cIMT thickening at baseline on incident hypertension. We have fully and comprehensively demonstrated the reliability of cIMT in identifying groups at high-risk for new-onset hypertension and extended previous research findings to the Chinese population.

CIMT is a convenient, low-cost, noninvasive indicator that can be widely used in clinical work and epidemiological surveys. From the perspective of pathophysiology, the increase in IMT can be divided into adaptive thickening and pathological thickening. The pathological manifestations of the former are smooth muscle cells, proteoglycans and collagen matrix hyperplasia, mainly related to age and blood pressure. The manifestations of pathological thickening are mainly the loss of smooth muscle cells and punctate calcification in the intima with deep lipid-rich and proteoglycan-rich formations [26]. Pathological intimal thickening is considered to be an early stage of atherosclerosis. The participants with thickened cIMT at baseline in our study presented an older age and higher BMI, rates of currently smoking and drinking, and greater prevalence of diabetes, dyslipidaemia and CVD. These observations suggest that this portion of the population may be more prone to early manifestations of atherosclerosis. Additionally, decreased vascular elasticity and increased vascular resistance caused by thickened IMT could further increase blood pressure and lead to the emergence of hypertension. Based on the results of our study, changes in arterial wall structure that preceded elevated blood pressure may be an important mechanism of hypertension.

Our study has certain limitations. The study focused on a community in Beijing, China, which limits the generalizability of our findings to other populations. Our research did not include inflammatory markers as adjustment factors for regression models. Out-of-office measurements are now recommended by most guidelines concerning the management of hypertension. Our study did not obtain data for ambulatory or home blood pressure measurements for the analysis. Future studies should supplement these data to further confirm the conclusion and focus on further elucidating the specific mechanisms by which vascular structures and functional changes affect blood pressure. However, it is necessary to assess whether preventive interventions in the population with thickened cIMT can effectively reduce the incidence of hypertension.

## Conclusion

In our study, based on a Chinese community population without hypertension at baseline, we identified that thickened cIMT has a strong association with new-onset hypertension.

## Data Availability

The data that support the findings of this study are available from the corresponding author upon reasonable request.

## Abbreviations

BMI: Body mass index
cIMT: Carotid intima-media thickness
CVD: Cardiovascular disease
DBP: Diastolic blood pressure
eGFR: Estimated glomerular filtration rate
SBP: Systolic blood pressure
SD: Standard deviation
